# Blindness Cured by the Extraction of Diseased Teeth

**Published:** 1874-01

**Authors:** 


### EDITORIAL, ETC.

---

*Blindness Cured by the Extraction of Diseased Teeth.*—Dr. Geo. S. Staples, of Corinth, Mississippi, sends us the following interesting case:

EDITOR JOURNAL.—*Dear Sir*—A case came under my notice some time since, that I have thought might prove of interest to the readers of the *Journal;* but have neglected to report it up to the present time. The case is as follows:

In passing through a neighborhood in the Eastern portion of this State in the spring of 1868, I stopped at a house where I met Mrs. S——, (aged about thirty-five.) She learned that I

was a dentist, and remarked that her teeth were very bad, and that she was anxious to have them extracted, but felt too nervous to do so. I then learned in the course of our conversation that she suffered a great deal from her eyes, and that they continued to grow worse.

I suggested that her teeth were the cause of the trouble with her eyes, (but never having met me before I think she had very little confidence in my judgment;) so she let her teeth remain, her eyes growing worse all the time, until about ten months after I met her she went totally blind, hardly being able to tell day from night. She called on some quack eye doctor, who happened to find her teeth were bad, and who advised her to have them extracted. She sent for me and had all her teeth extracted that were affected. She commenced immediately to recover her sight, and in a short time it was nearly as good as it ever was, and continues so up to the present time.

